# Supplementary material for: KEGG_Extractor: An Effective Extraction Tool for KEGG Orthologs
Source: Genes (Basel). 2023 Feb 1;14(2):386. doi: 10.3390/genes14020386 (PMC9956942; doi:10.3390/genes14020386)
Supplement: Supplementary file 1 [file genes-14-00386-s001.zip › genes-2042353-supplementary.pdf]

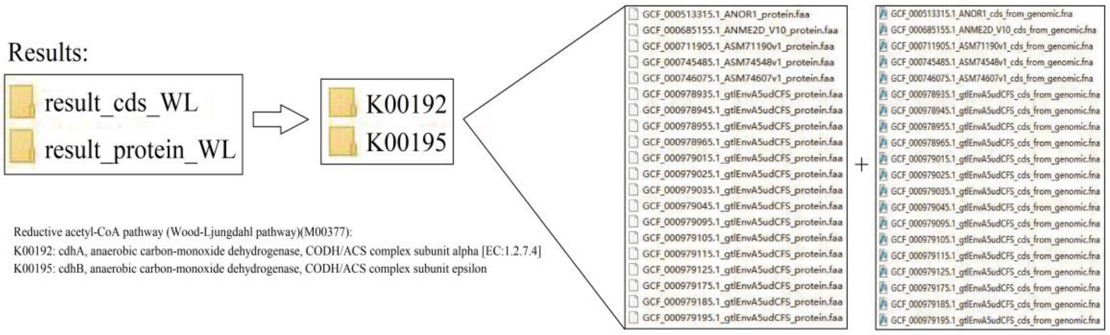

Supplementary file S1. Results of the tested twenty reference archaeal genomes implemented by the KEGG_Extractor tool.
